# Key factors predicting problem-based learning in online environments: Evidence from multimodal learning analytics

**DOI:** 10.3389/fpsyg.2023.1080294

**Published:** 2023-02-06

**Authors:** Xiang Wang, Di Sun, Gang Cheng, Heng Luo

**Affiliations:** ^1^Faculty of Education, Beijing Normal University, Beijing, China; ^2^Faculty of Humanities and Social Sciences, Dalian University of Technology, Dalian, Liaoning, China; ^3^Department of Information Technology, The Open University of China, Beijing, China; ^4^Engineering Research Center of Integration and Application of Digital Learning Technology, Ministry of Education, Beijing, China; ^5^School of Educational Information Technology, Central China Normal University, Wuhan, Hubei, China

**Keywords:** problem-based learning, peer engagement, learning process, multimodal learning analytics, online learning

## Abstract

Problem-based learning (PBL) has been used in different domains, and there is overwhelming evidence of its value. As an emerging field with excellent prospects, learning analytics (LA)—especially multimodal learning analytics (MMLA)—has increasingly attracted the attention of researchers in PBL. However, current research on the integration of LA with PBL has not related LA results with specific PBL steps or paid enough attention to the interaction in peer learning, especially for text data generated from peer interaction. This study employed MMLA based on machine learning (ML) to quantify the process engagement of peer learning, identify log behaviors, self-regulation, and other factors, and then predict online PBL performance. Participants were 104 fourth-year students in an online course on social work and problem-solving. The MMLA model contained multimodal data from online discussions, log files, reports, and questionnaires. ML classification models were built to classify text data in online discussions. The results showed that self-regulation, messages post, message words, and peer learning engagement in representation, solution, and evaluation were predictive of online PBL performance. Hierarchical linear regression analyses indicated stronger predictive validity of the process indicators on online PBL performance than other indicators. This study addressed the scarcity of students’ process data and the inefficiency of analyzing text data, as well as providing information on targeted learning strategies to scaffold students in online PBL.

## Introduction

Problem-based learning (PBL) is a pedagogical philosophy covering a multitude of practices and has been employed in different institutes and diverse domains in the last 50 years (Kilinska and Ryberg, 2019). PBL aims to educate students through the process of solving problems (Neville, 2009). In PBL, students are empowered with full autonomy to interact with others and use their skills and knowledge to develop a viable solution (Savery, 2006).

Given the popularity of the Internet, PBL has increasingly been carried out in online environments or blended settings. Technology-enhanced settings enable students to use various tools to perform tasks and solve problems, which leads to the generation of large amounts of data in the learning process (Unal, 2019). These data are very valuable for investigating in-depth PBL information, however, the data themselves cannot show anything without effective analysis. An emerging field addressing this challenge is learning analytics (LA), which has the capability to auto-analyze large amounts of data and presents the analysis directly to related stakeholders (Pan et al., 2020). Researchers believe that this can, in turn, empower educators to be more aware of students’ progress, assess their contributions based on evidence-based criteria, and identify patterns of low engagement and students at risk of failure (Foster and Siddle, 2020).

However, current applications of LA in PBL have not related LA results with specific PBL steps: Problem-solving performance and awareness were usually predicated by the overall LA without differentiation of the theoretical stages of PBL (Chen et al., 2019; Ludwig and Rausch, 2022). Additionally, text data generated from peer interaction are not mined effectively by LA methods. Recent reviews indicate that text mining and discourse analysis have not bee widely implemented and researched for educational purposes, compared to other analytic methods (Khalil and Ebner, 2016; Nkhoma et al., 2020). To identify students’ learning progress, problem-solving performance, and need for assistance, as well as to provide fair assessment and proper scaffolding, this study employed multimodal learning analytics (MMLA) based on machine learning (ML) to quantify the process engagement of peer learning based on text data, identify log behaviors, self-regulation, and other factors to predict online PBL performance.

## Literature review

### Problem-based learning

PBL is a student-centered pedagogy triggered by an ill-structured problem-solving scenario (Permatasari et al., 2019), in which students are enabled to participate in “learning by doing” actively and to develop transversal and lifelong learning skills (Sohmen, 2020). PBL originates from constructivist conceptions, which view learning as the active construction of knowledge that occurs through social interaction and dialogue among learners (Saqr and Alamro, 2019). The principal idea behind PBL is that the subject matter content and skills to be learned are organized around shared problems (Saqr et al., 2020). In PBL, students need to articulate the problem and then search, evaluate, construct, and share information which is then applied to a problem-solving situation in the real world (Neville, 2009). PBL thus helps students to improve their critical thinking, problem-solving ability, cognitive skills, and overall performance (Joshi et al., 2020). It is also an effective way to cultivate students to achieve 21st-century skills, such as being able to communicate and collaborate to solve complex problems, innovate in response to new demands and changing circumstances, and use technology to build new knowledge (Binkley et al., 2012).

Researchers summarize four key elements of PBL: the design of ill-structured learning problems, the role of the instructor as a facilitator, students’ self-regulation in the learning process, and peer learning to interact with others (Savery, 2006; Kilinska and Ryberg, 2019). The notion of ill-structured problems as the driving force for learning is a very central aspect of PBL (Kilinska and Ryberg, 2019). As opposed to presenting direct facts and conventional concepts in traditional instruction, complicated real-world problems are used in PBL to improve and promote student learning (Joshi et al., 2020). The role of the instructor during the process becomes that of facilitator to assist students to solve the problem (Horak and Galluzzo, 2017). Thus, students have to take the responsibility to be self-directed and self-regulated in their learning, which requires them to purposefully regulate their own cognitive, motivational, and emotional behavior, as well as that of others for optimal learning (Zimmerman, 2011). Peer learning and interaction are particularly meaningful in PBL. By working together in small groups, students are expected to actively communicate, share their expertise and previous knowledge, make joint decisions, and negotiate responsibilities, as well as to evaluate and modify the strategies of learning and group work through interactive dialogue (Hennessy and Murphy, 1999; Saqr et al., 2020).

PBL has been applied in multiple domains, and different models have been proposed across the world, such as the Alborg model with eight steps in Project Management; the Maastricht model with seven steps in Science, Healthcare, and Business; the Manchester model with eight steps in Medicine and Engineering; and the Samford model with seven steps in Business, Education, and Pharmacy, among others (Zotou et al., 2020). The details of the steps in these models are not the main part of the discussion here, but, generally, various models of PBL feature peer learning and four key steps for solving ill-structured problems: problem representation, solution development, making justifications, and monitoring and evaluating (Xun and Land, 2004).

With the rapid development of information technology and digital devices, online learning has become an acceptable educational format throughout the world. As a typical pedagogy, PBL has increasingly been carried out in online environments. Online discussion is a very important way to support PBL, especially peer learning, in online settings (Saqr and Alamro, 2019); it requires students to engage in active discussions in two types of dialogical spaces: content and relational spaces. The goal in the content space is to acquire a deeper understanding of the knowledge and skills in the domain by collecting information, discussing concepts, and proposing solutions to the problem; the relational space deals with interpersonal relationships and interactions among collaborators (Slof et al., 2010; Saqr and Alamro, 2019). There is overwhelming evidence of the value of PBL; however, offering students an ill-structured problem does not directly translate to effective interactions and high performance. PBL requires scaffolding by instructors, coordination of peer learning, and active engagement of students in a stimulating environment, which calls for a mechanism to monitor the efficiency of engagement and design a data-driven intervention that supports effective PBL (Pan et al., 2020). LA is an interesting emerging field that could address these challenges.

### Learning analytics

LA is concerned with the measurement, collection, analysis, and reporting of data about learners and their contexts, for purposes of understanding and optimizing learning and the environments in which it occurs (Siemens, 2013). The popularity of online learning, the challenge of extracting value from educational big data, and the demand to improve performance are the three driving forces in the emergence of LA (Ferguson, 2012). The data used in LA are mainly gathered through monitoring students’ activity in online learning platforms (e.g., access to resources, logins, textual input) and materials from other various tools, techniques, or environments (e.g., forums, blogs, interactive whiteboards, social sites, and libraries) (Kilinska and Ryberg, 2019). LA methods are based on educational data mining, which includes relationship mining, prediction, modeling the user’s knowledge domain, personalization and adaption, and structure discovery and analysis, as well as traditional evaluation and monitoring (Siemens, 2013). The LA domain can thus accumulate as much data as possible and enable stakeholders to understand the learning process, identify students’ knowledge and skills, detect students’ weaknesses and misconceptions, evaluate the assessment’s efficiency, and ultimately improve learning (Zotou et al., 2020).

However, most LA research focused more on clickstream data than other types of data to measure learning and instruction, with few considering psychological characteristics and the text content generated by students, thereby making LA research seem like observational reports without enough learning and instructional guidance for practices (Tsai et al., 2020). Recently, MMLA has emerged as an exciting field within the LA domain, which focuses more on the diversity of data and methods in the research process than LA before (Di Mitri et al., 2018; Emerson et al., 2020).

MMLA builds upon multimodal human interaction, educational data mining, learning sciences, and many other fields to capture the complexity of learning through data-intensive approaches (Spikol et al., 2018). First, MMLA captures multimodal data from bodily movements, face tracking, affective sensors, hardware and software log files, and user and research-generated data (e.g., discourse data). Further, it focuses on developing a better understanding of the complexity of learning through advances in high-frequency multimodal data capture, signal processing, ML techniques, and statistical methods (Ochoa and Worsley, 2016). We, therefore, believe that MMLA may offer an opportunity to capture different insights about learning in PBL to provide effective support to help students achieve good performance. In this study, since LA were collected from a semester-long course, it was infeasible to incorporate certain multimodal data (e.g., emotion, motion, and biostats) generated from sensor-based technologies, despite their proven value as MMLA (Sharma et al., 2019). Consequently, the MMLA used in the current study mainly comprised three types: psychological characteristics, activity/engagement data, and student-generated text content.

### Research on integrating LA into PBL

Although many researchers have proved and introduced LA or MMLA into PBL area, the relevant research is still limited (Zotou et al., 2020). So far, two kinds of attempts have been made in the pursuit of integrating LA with PBL. One is concerned with LA as a tool in learning platforms to collect learning and instructional information, generate relevant statistical data, and provide insights into the exchange of information for stakeholders within the learning platforms. For example, Hogaboam et al. (2016) investigated the use of LA tools to support instructors in facilitating an online PBL workshop for medical students (Hogaboam et al., 2016). The researchers collected multimodal data from videos, discussions, and whiteboards through PBL, and then built an LA dashboard to visualize student performance with a scrollable news feed, a graph of the discussion, and a word cloud. Similarly, the study by Spikol et al. (2018) also focused on applying LA to multimodal data deriving from diverse sensors (computer vision, user-generated content, and data from the learning objects) during PBL and presented an LA dashboard to visualize the results and help educators determine whether groups are performing well (Spikol et al., 2018). Triantafyllou et al. (2018) developed a platform in Moodle by employing LA to monitor students’ learning pathways during PBL group work (Triantafyllou et al., 2018). This provided a communication and information channel between project supervisors and students, as well as between students belonging to the same group.

The other attempt has been to use LA as a research method to investigate academic topics in PBL, where the main attention is on the prediction of student performance. Tempelaar et al. (2014) proposed a dispositional LA infrastructure that combines learning dispositions data with student engagement/activity data from the learning management system, as well as data extracted from computer-assisted formative assessments (Tempelaar et al., 2014). The results showed that computer-assisted formative assessments were the best predictor of academic performance, while basic data from the learning management system did not substantially predict learning. Saqr et al. (2020) focused on interactivity relationships in online PBL, and employed social network analysis (SNA) to investigate which factors can improve the monitoring, facilitation, and prediction of student performance (Saqr et al., 2020). They found that SNA analysis can enable the prediction of performance in groups and support students with limited participation and interactions.

However, these studies, whether regarding LA as a tool or a method, did not relate the LA results and findings with specific PBL steps or pay sufficient attention to the interaction in peer learning. More particularly, they have not targeted the analysis of text data generated from peer interaction. Although several studies have used SNA to investigate the interactivity relationship in PBL and shown that SNA can help to map the patterns of interactions and quantify the structural properties of learning groups (Dado and Bodemer, 2017; Saqr and Alamro, 2019), all of these efforts are limited in the relational space and only leverage the content space of interaction to a limited degree.

The lack of in-depth analysis of interaction content imposes many challenges in detecting the state of students’ knowledge, skills, and affection, and providing them with timely and proper facilitation. Students may suffer from poor cognition, lack of skills, low motivation, or self-suspicion. If these challenges are not effectively addressed, students may be disengaged from learning, inactive in collaboration, have low performance, and even withdraw from learning. However, with the current instrumentations, much time and effort are required for human beings to code text from interactions, interviews, or surveys, which means such methods cannot deliver automated analysis of and effective insights about the learning process to educators (Saqr and Alamro, 2019). Fortunately, after decades of development, the use of text analysis—or natural language processing (NLP)—has been attempted in education, and researchers have increasingly targeted text data. Text can easily be gathered from face-to-face and online activities, which constitutes one of the most promising modalities for MMLA and will likely accelerate discourse-based research, as well as opening up new possibilities for large-scale analysis of open-ended text corpora in education (Blikstein and Worsley, 2016).

## Research methodology

### Research questions

To address the gap in PBL research and take advantage of multimodal data, especially text data, this study proposes NLP-and ML-enhanced strategies for PBL in online settings. Based on the key elements of PBL (especially peer learning), the goal of this exploratory MMLA research is to better process, interpret, and present various student-generated data to support the online PBL process. Specifically, this study seeks to try effective ways to present content-and process-based indicators to facilitate PBL activities in online settings. Demographic variables such as gender and prior knowledge were also included in our analysis as potential influencing factors, and we aimed to control for their cofounding impact on students’ PBL performance. In particular, the following research questions guided our investigation:

RQ1. How do gender differences and prior knowledge influence students’ PBL performance in online settings?

RQ2. How does students’ self-regulation influence their PBL performance in online settings, holding gender differences and prior knowledge constant?

RQ3. How does students’ peer learning engagement (i.e., log behaviors, and process engagement in problem-solving steps) predict their PBL performance in online settings, controlling for gender differences, prior knowledge, and self-regulation?

### Participants and pedagogical design

The participants of this study were 104 fourth-year students in an online course on social work and problem-solving in 2021 at a Chinese university. This course was conducted on Moodle and aimed to introduce social work theories and train students’ problem-solving skills based on three social work cases with ill-structured problems. PBL of each case in this course was designed based on the four-step problem-solving model by Xun and Land (2004), which includes: (a) problem representation, (b) generating and selecting solutions, (c) making justifications, and (d) monitoring and evaluating goals and solutions (Xun and Land, 2004). The question prompts of each step were listed in the online discussion module of Moodle to facilitate students’ PBL process. The primary goal was to assist students in going through the four steps of the problem-solving process based on the repeated practice of the three cases with ill-structured problems. Finally, we expected them to effectively understand the relevant social work theories and confidently resolve real social work problems without the instructor’s facilitation at the end of the semester. The participants aged from 22 to 24, with a mean age of 23.04.

The main part of the 16-week course process was peer learning based on online discussion. Participants were randomly assigned into 13 groups consisting of eight members. In the online discussion, based on each case, the instructor provided students with specific-domain question prompts for the four steps. Based on these prompts, students could initiate their group learning, organize their thoughts, share their ideas, and communicate with peers. Further, they could improve self-regulation to construct meaningful social work plans based on personal knowledge and problem-solving skills.

The research procedure and instruments of the present study were reviewed and approved by the Institutional Review Board of Central China Normal University (CCNU-IRB-201909021, approved on 2019/09/16). Students were made aware that their participation in the research study was completely voluntary and they had the right to withdraw from the study at any time without penalty. All their personal identifiable information would remain confidential and would not appear in any publications or presentations. The participants provided their written informed consent to participate in this study.

### Research design and data

**Figure 1:**
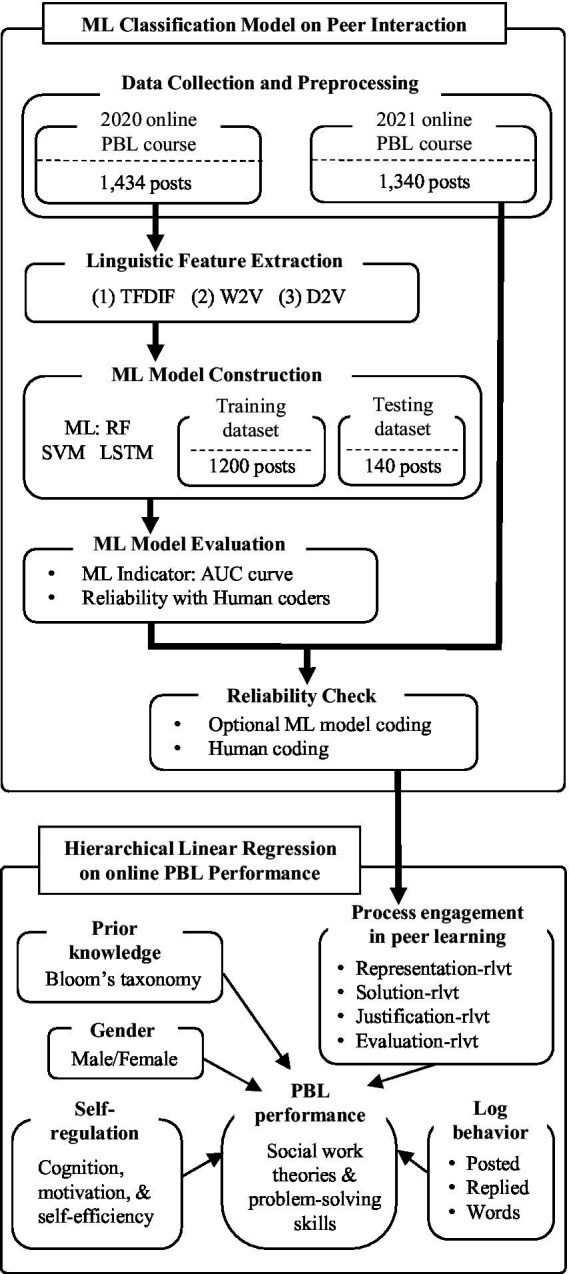
Research process.

This study employed the MMLA method to investigate students’ learning process in online PBL. The whole MMLA deployment process includes data collection and preprocessing, linguistic feature extraction, ML classification model construction, model performance evaluation, and building the hierarchical linear regression models. The details are shown in Figure 1 and described in the section on Data Analysis.

There are two main parts: the first is “ML Classification Model on Peer Interaction,” which focuses on online discussion to identify peer learning engagement in each step through the problem-solving process. The discussion messages posted by students in the 2020 online PBL course were coded by human coders (the course instructor and a teaching assistant) and were labeled with their relevance with the course topic. We used human coding results to train and evaluate ML algorithms to automatically label the messages posted in the 2021 online PBL course. The second is “Hierarchical Linear Regression on online PBL Performance,” which involves building hierarchical linear regression models to identify key factors predicting students’ PBL performance in online learning and evaluating their importance by measuring their predicting capacity.

As mentioned above, the key principle affecting PBL is peer learning. Student discourse in online discussions forms the key data to identify the peer learning engagement of each step through PBL. A total of 1,434 online discussion posts from the 2020 semester and 1,340 messages from the 2021 semester were collected. The messages from 2020 were used to train and test the ML classification models based on NLP and ML algorithms. The optional model then categorized the 1,340 posts from 2021 into course-relevant and course-irrelevant data; this division formed the data used in the final hierarchical linear regression models on PBL performance. Moreover, students’ log behaviors in Moodle, including the number of messages a student posted, and replied to, and the total words of messages were also collected as another kind of data indicating peer learning.

We also collected students’ gender and self-regulation information through questionnaires, as well as prior knowledge from students’ reports. The final PBL performance in this study was a combination of social work theories and problem-solving skills. Age is a demographic factor always related to learning performance; however, in this study, except for 7 students 24 years old and 3 students 22 years old, 94 of the 104 students were 23 years old, which means over 90% of students were of the same age. Therefore, we did not include age as a key influential factor in this study.

### Instrument

#### Prior knowledge

At the beginning of the course, without any instruction on social work knowledge and problem-solving skills, students were required to write a design report to try to resolve the problems in the first case based on their prior knowledge. Then, two experts graded students” reports from 1 to 6 based on Bloom’s taxonomy of cognitive levels, which includes recognition, understanding, analysis, application, synthesis, and evaluation. With acceptable inter-rater reliability (Spearman’s Rho>0.7), the average ratings of the two experts were used as the final scores of students’ prior knowledge.

#### Self-regulation

Like prior knowledge, student’s self-regulation tendency and skills are considered as important personal trait that influences online learning performance and has a reciprocal relationship with online learning engagement, motivation, and interaction (Cho and Kim, 2013; Zheng et al., 2018; Miao and Ma, 2022). We developed a questionnaire to measure self-regulation that includes three constructs: cognition, motivation, and self-efficiency; Cronbach’s Alpha is 0.856. Cognition consists of eight subscales adopted from the Community of Inquiry (CoI) framework (Arbaufvgh et al., 2008). Motivation is adapted from the scale developed by Lin et al. (2020). Self-efficiency included seven items adapted from the instrument developed by Artino and McCoach (2008).

#### Peer learning engagement

As mentioned above, we collected two kinds of data relevant to peer learning engagement. One was the process engagement of four problem-solving steps based on the classified discourse data through the optional ML classification model. The other was the log behaviors including the number of messages each student posted, replied to, and the total words of his/her messages.

#### PBL performance

In this study, PBL performance was a combination of social work theories and problem-solving skills. The full score of students’ PBL performance was 100 points in two parts: multiple-choice questions on social work theories (20 points), and a final report on resolving an ill-structured social work problem by individual students themselves (80 points). The multiple-choice questions were adapted from the test banks of the National Graduate Entrance Exam, which aimed to examine students’ recall and comprehension of social work knowledge taught in the course. The final report assignment was designed and graded by the course instructor, which aimed to test students’ ability to apply the learned knowledge into solving authentic social work problems. The quality of final report was evaluated by four criteria: completeness of analysis, diversity of perspectives, justification of the final solution, and overall logic of reasoning.

## Data analysis and results

Figure 1 illustrates the data analysis based on the two main parts of this study, with details described below.

### Discourse data collection and preprocessing

To train the ML classification models, we collected 1,434 posts in online discussion data from students enrolled in this course in the 2020 academic year. These discourse data were dichotomously coded 1 as course-relevant, or 0 as course-irrelevant by the course instructor and teaching assistant. The Kappa value was near 0.93, which means a high consistency between coders. Among the coding results, 75% of the data were course-relevant.

### Linguistic feature extraction

Three linguistic feature extraction (LFE) methods were employed in this study, including term frequency and inverse documentation frequency (TFIDF), Word2Vec (W2V), and Doc2Wec (D2V). TFIDF identifies the contribution of a word by calculating the frequency at which the word appears in the text and the whole corpus (Guo and Tao, 2016). W2V transforms each word into a multidimensional vector space; the distance between the vectors represents the similarity between the words (Lilleberg et al., 2015). D2V is an extension of W2V, extending the learning of the embeddings from words to word sequences, and agnostic to the granularity of the word sequence—it can be a word n-gram, sentence, paragraph, or document (Le and Mikolov, 2014).

### ML classification model construction

Three ML algorithms were employed: random forest (RF), support vector machine (SVM), and long short-term memory (LSTM). The 1,434 online discussion posts from 2020 were randomly split into two subsets: 1,300 posts for training and 134 posts for testing. Based on the three LFE methods and three ML algorithms, nine different ML classification models were constructed.

### Model performance evaluation

Generally, several common indicators are used to evaluate ML model performance, including accuracy, precision, recall, specificity, F1, and AUC (Powers, 2020). AUC represents the probability that the ML model can correctly classify randomly chosen course-relevant posts. AUC was adopted in this study because it can provide impartial evaluation even when the classification of the data is imbalanced. Based on the human coding results, 75% of messages were course-relevant and 25% were course-irrelevant. Therefore, AUC was the indicator chosen for ML model performance in this study. We evaluated the performance of the nine ML classification models through AUC with a 10-fold cross-validation method to prevent overfitting. The AUC values are listed in Table 1, and a larger AUC means the model is more powerful in classification.

**Table 1:** ML model performance evaluation.

ML algorithm	Linguistic feature extraction	AUC	Kappa
RF	TFDIF	0.867	0.801
	W2v	0.861	0.789
	D2V	0.863	0.791
**SVM**	TFDIF	0.846	0.769
	W2v	0.876	0.810
	**D2V**	**0.880**	**0.816**
LSTM	TFDIF	0.852	0.734
	W2v	0.903	0.749
	D2V	0.891	0.760

Bold values indicate the optimal ML classification model based on a balanced performance indicated by AUC and Kappa values.

We also employed the Kappa value to identify reliability and compare the consistency between the coding results from the ML classification models and those made by human coders. The details are also shown in Table 1; a higher Kappa indicates better consistency between the model results and human coding. To balance the efficacy of the ML classification model and consistency, we made a trade-off between AUC and Kappa to choose D2V_SVM as the final ML classification model to analyze the 1,340 online discussion posts from the 2021 academic year.

### Reliability checking of data from 2021

The D2V_SVM model categorized the 1,340 posts from 2021 into course-relevant and course-irrelevant. We also randomly chose 100 posts to be coded by human coders. The Kappa value between the ML model coding and the human coding was 0.82, which indicates acceptable consistency. To gain an in-depth investigation of peer learning engagement in each problem-solving step, we related each courser-relevant message to a certain problem-solving step based on its log record. There were a total of 1,132 relevant messages distributed among the four PBL steps: Representation-relevant (Representation-rlvt, *n* = 376), Solution-relevant (Solution-rlvt, *n* = 316), Justification-relevant (Justification-rlvt, *n* = 275), and Evaluation-relevant (Evaluation-rlvt, *n* = 165). A larger number of relevant messages generated by students indicates greater relevance of peer interaction and discourse to the PBL task, and thus shows a higher level of peer learning engagement during the online PBL course.

### Hierarchical linear regression analyses on problem-solving performance

Based on the instructional settings and the preparation of the ML classification model, we finally selected the possible factors that predict students’ problem-solving performance in online PBL, including gender, prior knowledge, self-regulation, three log behavior variables, and peer learning engagement during the four steps of PBL as measured by the relevance of messages posted in each step. The descriptive statistics are shown in Table 2.

**Table 2:** Descriptive statistics of variables (*N* = 104).

Variable	Mean	*SD*	Min	Max
PBL performance	75.82	11.409	42.41	95.19
Gender	0.37	0.485	0	1
Prior knowledge	3.406	0.466	3.0	5.0
Self-Regulation	3.754	0.457	2.78	5.00
Posted	9.30	4.703	1	25
Replied	4.28	1.717	3	9
Words	6,025	4,921	515	30,691
Representation-rlvt	3.62	0.981	2	6
Solution-rlvt	3.04	0.862	1	5
Justification-rlvt	2.65	0.926	1	5
Evaluation-rlvt	1.59	0.76	1	4

Five hierarchical linear regression models were built to investigate the unique and combined influences of these variables on online PBL performance (See Table 3). The scores for the variance inflation factors (VIFs) in each regression model were lower than 2.5, which indicates there were no collinearity issues among the independent variables in this study. The results of Model 1 showed that individual differences in gender and prior knowledge accounted for 0.057 of variance in online PBL performance, and prior knowledge was the only significant predictor (*β* = 0.241, *p* = 0.016). In Model 2, after adding the self-regulation factor, 20% more variance in online PBL performance was explained (*ΔR*^2^ = 0.204, *p* < 0.001), holding the Model 1 variables constant. This result suggested that students’ self-regulation played a significant role in online PBL (*β* = 0.452, *p* < 0.001). The *R*^2^ and adjusted *R*^2^ of Model 2 were 0.261 and 0.239.

**Table 3:** Hierarchical linear regression analysis to predict PBL performance.

	Model 1					Model 2				
	*B*	*SE*	*β*	*p*	*VIF*	*B*	*SE*	*β*	*p*	*VIF*
Intercept	55.622	8.380		0		13.088	11.044		0.239	
Gender	0.331	2.303	0.014	0.886	1.016	−0.142	2.052	−0.006	0.945	1.018
Prior knowledge	5.895	2.395	0.241	0.016	1.016	5.997	2.131	0.245	0.006	1.016
Self-regulation	–	–	–	–	–	11.285	2.161	0.452	0.000	1.002
*R*^2^ (Adjusted *R*^2^)	0.057 (0.039)					0.261 (0.239)				
*ΔR* ^2^	0.057					0.204				
*F*	5.044*					11.650**				
	Model 3A					Model 3B				
	*B*	*SE*	*β*	*p*	*VIF*	*B*	*SE*	*β*	*p*	*VIF*
Intercept	29.368	9.230		0.002		9.895	8.820		0.265	
Gender	0.663	1.631	0.028	0.685	1.030	−0.733	1.593	−0.031	0.646	1.046
Prior knowledge	2.263	1.805	0.093	0.213	1.167	2.014	1.728	0.082	0.247	1.139
Self-regulation	7.371	1.808	0.295	0	1.122	8.028	1.739	0.321	0	1.106
Posted	0.490	0.209	0.202	0.021	1.597	–	–	–	–	–
Replied	0.010	0.458	0.002	0.982	1.018	–	–	–	–	–
Words	0.001	0.000	0.446	0	1.680	–	–	–	–	–
Representation-rlvt	–	–	–	–	–	3.321	0.973	0.286	0.001	1.598
Solution-rlvt	–	–	–	–	–	3.918	1.159	0.296	0.001	1.753
Justification-rlvt	–	–	–	–	–	0.283	1.056	0.023	0.79	1.677
Evaluation-rlvt	–	–	–	–	–	2.841	1.199	0.189	0.02	1.455
*R*^2^ (Adjusted *R*^2^)	0.552 (0.524)					0.584 (0.553)				
*ΔR* ^2^	0.291					0.323				
*F*	19.722**					19.043**				
	Model 4									
	*B*		*SE*		*β*		*p*	*VIF*		
Intercept	17.848		7.969				0.028			
Gender	0.158		1.352		0.007		0.907	1.06		
Prior knowledge	1.252		1.531		0.051		0.416	1.256		
Self-regulation	5.820		1.517		0.233		0.000	1.182		
Posted	0.673		0.186		0.277		0.000	1.88		
Replied	−0.011		0.396		−0.002		0.978	1.139		
Words	0.000		0.000		0.185		0.030	2.245		
Representation-rlvt	2.225		0.868		0.191		0.012	1.791		
Solution-rlvt	2.962		0.995		0.224		0.004	1.816		
Justification-rlvt	0.635		0.908		0.051		0.487	1.744		
Evaluation-rlvt	2.683		1.089		0.179		0.016	1.688		
*R*^2^ (Adjusted *R*^2^)	0.713 (0.682)									
*ΔR* ^2^	0.161(M4 vs. M3A)/0.130(M4 vs. M3B)									
*F*	22.899**									

*indicates *p* < 0.05, ** indicates *p* < 0.001.

In Model 3A and Model 3B, we examined the respective influences of peer learning of three log behaviors and peer learning engagement in four problem-solving steps beyond Model 2. We observed that adding three log behaviors in Model 3A accounted for an additional 0.291 of variance explained in online PBL performance (*ΔR*^2^ = 0.291, *p* < 0.001), holding the Model 2 variables constant. The result demonstrated that students who frequently posted messages had better academic performance (*β* = 0.202 *p* = 0.021), as well as students with more message words (*β* = 0.446, *p* < 0.001). The *R*^2^ and adjusted *R*^2^ of Model 3A were 0.552 and 0.524. Adding peer learning engagement in four problem-solving steps to Model 3B accounted for an additional 0.323 of variance explained in online PBL performance beyond Model 2 (*ΔR*^2^ = 0.323, *p* < 0.001). This result indicated that students who engaged more in the problem representation step were more likely to have better online PBL performance (*β* = 0.286, *p* = 0.001), as did students who engaged more in the problem solution step (0.296 0.001) and the problem evaluation step (*β* = 0.189, *p* = 0.02). The *R*^2^ and adjusted *R*^2^ of Model 3B were 0.584 and 0.553, respectively.

Model 4 was the integrated model, investigating the combined influences of all of the study variables on online PBL performance. The *R*^2^ and adjusted *R*^2^ of Model 4 were 0.713 and 0.682, respectively. The results showed that students’ prior knowledge no longer significantly predicted performance in online PBL (*β* = 0.051, *p* = 0.416). Students’ self-regulation was still significant in predicting performance in online PBL (*β* = 0.233, *p* < 0.001). However, peer learning engagement (log behaviors and process engagement) is highlighted as the main explanation of online PBL performance. Model 4 explained significantly more of the variance than Model 3A (*ΔR*^2^ = 0.161, *p* < 0.001) and Model 3B (*ΔR*^2^ = 0.13, *p* < 0.001). However, the model comparison results also suggested that process engagement in peer learning (classified by ML models as course-relevant to each problem-solving step) had stronger predictive validity than peer learning of log behavior in explaining online PBL performance, holding all other variables constant.

## Discussion

### Influence of gender and prior knowledge on online PBL performance

To answer the first research question, Model 1 indicated that students’ prior knowledge was a significant indicator of final PBL performance in online settings. Previous studies have illustrated that prior knowledge could reduce cognitive load and lead to better learning engagement, so it is one of the powerful influential factors in determining final learning achievement (Dong et al., 2020; van Riesen et al., 2022). The result of the current study is in line with previous findings and especially highlights that prior knowledge is influential on students’ performance in online PBL settings. Focusing on this study, among the cognitive levels in Bloom’s taxonomy, most students’ prior knowledge was at level 3, analysis. This means that students were short of enough knowledge and skills in application, synthesis, and evaluation to resolve ill-structured social work problems. However, such higher-level cognition abilities are essential in PBL. Therefore, the course instructor designed specific question prompts to scaffold students in the problem-solving process. We would like to insist that understanding the state of students’ prior knowledge is very important in PBL to provide students with appropriate and adequate facilitation.

### Influence of student self-regulation on online PBL performance

For the second research question, the result of the current study was consistent with general experience and our expectations: in Model 2, students’ self-regulation was validated as a positive and powerful predictor of online PBL performance. Due to the COVID-19 pandemic, online learning has become inescapable support to traditional face-to-face instruction, which is more popular in a variety of educational institutes. At present, online learning is regarded as a routine to effectively carry out learning and instruction in case of unexpected events. Self-regulation or self-direction is always a research focus in the area of online learning. Because of the separation of time and space in online learning, improving and sustaining students’ self-regulation ability is a complex topic that has attracted the attention of many researchers. As a particular format of online learning, online PBL is triggered by ill-structured problems and requires students to fix the problem with higher-order thinking and skills; this means online PBL sets higher requirements for students’ self-regulation in the problem-solving process. Therefore, helping students to regulate their attention on cognition, motivation, and affection through the problem-solving process can enhance their learning performance (Zimmerman, 2011). In this study, the instructor provided four steps to clarify the process of solving ill-structured problems. The study findings suggested that such process-oriented strategies scaffolded students to regulate their learning, communicate with their peers, and direct their attention to solving problems.

### Influence of peer learning engagement on online PBL performance

To answer the third research question, Models 3A, 3B, and 4 were developed. In Model 3A, after controlling for student gender, prior knowledge, and self-regulation, three log behaviors of peer learning showed a significant impact on problem-solving performance in online PBL. Messages posted and the total words of messages were, in particular, significant influential factors on problem-solving performance. The number of messages a student replied to was not a significant predictor of problem-solving performance. After checking the relevant data, we found that students posted almost twice as many messages as they replied to. We speculated that students were more used to sharing their thoughts with their peers than answering others’ questions. This should remind instructors to pay attention to encouraging students to help their peers in group learning and improve relevant abilities of their own.

Similarly, in Model 3B, after controlling for the same variables as in Model 3A, peer learning engagement in four steps of problem-solving significantly predicted online PBL performance. The results were in line with the literature, which has found that those students who actively communicated with peers, shared their knowledge, discussed problems, negotiated solutions, and evaluated strategies would achieve better performance (Hennessy and Murphy, 1999). However, we found that in the four steps of the problem-solving process, only engagement in Representation, Solution, and Evaluation were significant predictors of problem-solving performance. Justification did not exert a powerful influence. We would like to clarify that these results do not mean that Justification is not an important factor for problem-solving performance. On the contrary, it might imply that students need to improve their knowledge and skills to justify their solution plans for ill-structured problems.

In Model 4, the integrated Model, we found that students’ prior knowledge was not a significant predictor of PBL performance; students’ self-regulation, log behaviors of posted and messages words, and process engagement (Representation, Solution, and Evaluation) were still the significant predictors, especially the powerful factors of process engagement. This indicated that, with adequate coding consistency with human coders, employing the ML classification model to identify the dynamic engagement of the PBL process can mine in-depth information throughout the entire semester than using psychometric measurements or human coding. In sum, the pedagogical integration of four-step online PBL with MMLA models can provide a comprehensive understanding of online PBL experience regarding gender, prior knowledge, self-regulation, and peer learning. It also supported the notion that ML-based LA methods can be used as an alert or diagnostic module to provide in-depth information and facilitate students in moving through different online PBL phases.

## Conclusion and limitations

This study constructed multimodal learning analytical models to investigate how peer learning engagement, especially dynamic process engagement in Representation, Solution, Justification, and Evaluation, along with self-regulation affects problem-solving performance under online PBL settings, holding gender and prior knowledge constant. The results revealed that self-regulation, messages posted, message words, process engagement in Representation, and Evaluation were predictive of online PBL performance. ML classification models were also built and evaluated to classify discourse data in online discussions. The optional model effectively and objectively identified peer learning engagement in different problem-solving steps in online PBL contexts and indicated stronger predictive validity of process indicators on online PBL performance than other indicators.

The study findings may contribute both theoretical and practical improvements to online PBL. Concerning theory building, this study adds to the literature that the process engagement of peer learning in online problem-solving can be incorporated into an integrated MMLA model to predict online PBL performance, which is a response to the scarcity of studies tracing students’ progress in PBL (Zotou et al., 2020). Regarding practical implications, this study illustrates that ML classification models based on NLP can be trained effectively to identify process engagement from discourse data instead of conducting intensive human coding; the MMLA model can thus be applied to monitor and detect students with poor peer learning engagement through the PBL process. The MMLA model’s predictions can help stakeholders to design strategies, make decisions, and conduct evaluations to foster students’ problem-solving abilities in online settings.

This study shows that gender does not have an impact social work problem-solving performance in online learning, but prior knowledge is the basis and start for further learning. We, therefore, suggest that before or at the beginning of the online PBL, students should have a pre-test to illustrate the state of their knowledge and skills relevant to the learning topic, which can help the instructors to set up effective scaffolds. Throughout the process of online PBL, instructors can take advantage of ML, MMLA, or other technology to monitor and evaluate students’ self-regulation and peer learning engagement, and then provide personalized facilitation to improve student performance.

There are, however, some limitations to this study. First, the participants in this study were drawn from only one of the social work courses at the undergraduate level, which might not represent students in other disciplines. Second, we only trained and tested limited NLP methods and ML classification algorithms, and the sample size was not very large. In the future, we would like to develop more ML classification models as well as further improve reliability. Third, although the process engagement based on discourse data was coded into course-relevant or course-irrelevant, and linked to the four problem-solving steps, the discourse may be coded into multiple classes in the future to investigate more in-depth information from the valuable data. Finally, as PBL and online learning both rely on self-regulation—especially intrinsic motivation in learning—future research could include more multimodal data from various learning settings, such as social media, intelligent agents, online platforms, onsite classrooms, or face-to-face environments, to integrate self-regulation and peer learning to foster a comprehensive MMLA model of online PBL.

## Data availability statement

The raw data supporting the conclusions of this article will be made available by the authors, without undue reservation.

## Ethics statement

The research procedure and instruments of the present study were reviewed and approved by the Institutional Review Board of Central China Normal University (CCNU-IRB-201909021, approved on 2019/09/16). The participants provided their written informed consent to participate in this study.

## Author contributions

DS and HL: conceptualization and writing–review and editing. XW and DS: methodology, investigation, and writing–original draft preparation. DS and GC: formal analysis. HL and GC: resources. DS and GC: project administration. DS: funding acquisition. All authors have read and agreed to the published version of the manuscript.

## Funding

This work was funded by Research on Intelligent Big Data and Community Peacebuilding, Grant of “Social Science Foundation of Liaoning Province in China” (L21BSH002).

## Conflict of interest

The authors declare that the research was conducted in the absence of any commercial or financial relationships that could be construed as a potential conflict of interest.

## Publisher’s note

All claims expressed in this article are solely those of the authors and do not necessarily represent those of their affiliated organizations, or those of the publisher, the editors and the reviewers. Any product that may be evaluated in this article, or claim that may be made by its manufacturer, is not guaranteed or endorsed by the publisher.
